# Unlocking New Treatment Horizons for Celiac Disease: PRKCD Revealed as a Promising Target through Mendelian Randomization

**DOI:** 10.2174/0118715303410531250815124524

**Published:** 2025-08-29

**Authors:** Jie Zhou, Yixin Xu, Haitao Wang, Kun Wang, Chao Chen

**Affiliations:** 1 Department of Gastrointestinal Surgery, The Wujin Hospital Affiliated with Jiangsu University, Changzhou, China;; 2 Department of Gastrointestinal Surgery, The Wujin Clinical College of Xuzhou Medical University, Changzhou, China;; 3 Department of Gastrointestinal Surgery, The Third Affiliated Hospital of Soochow University, Changzhou, China

**Keywords:** Tight junctions, protein kinase C, claudins, druggable genes, single nucleotide polymorphism, intestinal barrier

## Abstract

**Introduction:**

Celiac Disease (CeD) is a serious, lifelong autoimmune condition. There remains a significant unmet medical need for effective pharmacological treatments for CeD.

**Methods:**

We utilized summary statistics for 2,888 druggable genes from the eQTLGen Consortium and the FinnGen Consortium for CeD. In our Mendelian Randomization (MR) analysis, we identified genes associated with CeD that had a false discovery rate (FDR) < 0.05 using the Inverse Variance Weighted (IVW) method. To enhance the reliability of the results, we validated them through colocalization analysis and Summary-data-based Mendelian Randomization (SMR) analyses.

**Results:**

Through our analysis, we identified 18 druggable genes with a causal relationship to CeD under an FDR < 0.05. Subsequent colocalization and SMR analyses highlighted the PRKCD gene as a potential therapeutic target for CeD (IVW method: Odds Ratio 1.319, 95% Confidence Interval 1.182-1.471, *P =* 6.85E-07, FDR = 0.002). Additionally, these results have passed horizontal pleiotropy tests, heterogeneity analysis, and leave-one-out sensitivity analysis.

**Discussion:**

The identification of PRKCD as a therapeutic target represents a significant advancement in addressing the unmet medical need for CeD treatment. However, the hypothesis that PRKCD contributes to CeD pathogenesis by regulating tight junction proteins and altering intestinal barrier function requires further experimental validation in future studies.

**Conclusion:**

Our study is the first to identify the PRKCD gene as a potential therapeutic target for treating CeD, providing new insights into the treatment of CeD and guiding the development of corresponding therapeutic drugs.

## 1 INTRODUCTION

Celiac disease (CeD) is a chronic autoimmune disorder of the small intestine, triggered by the ingestion of gluten in genetically predisposed individuals [1]. The pathogenesis involves a complex interaction between genetic susceptibility and environmental factors, primarily gluten [2]. Chronic mucosal damage and inflammation lead to malabsorption of nutrients, including deficiencies in calcium, vitamin D, iron, vitamin B12, folate, and zinc [3, 4]. Over the past two decades, CeD has become a significant global public health issue [5].

Currently, the U.S. Food and Drug Administration has not approved any drugs for the treatment of CeD [6]. So far, a gluten-free diet is the only known viable treatment option. However, avoiding products containing gluten is very challenging in practice, mainly due to the risk of cross-contamination. Products labeled as “gluten-free” are allowed to contain up to 20 ppm of gluten [6]. In reality, maintaining a completely gluten-free diet is difficult. Despite adopting a gluten-free diet (GFD), about half of the patients still experience villous atrophy [7, 8]. This persistent villous atrophy is associated with symptoms such as nausea, diarrhea, abdominal pain, and bloating, and it also increases the risk of lymphoproliferative malignancies [9, 10]. Therefore, it remains crucial to identify potential drug targets that could treat or delay the onset or progression of CeD. Human genetics research has been widely applied in the field of drug development for various complex diseases. Drug targets supported by human genetic evidence are more likely to succeed in clinical trials [11, 12]. For example, in GWAS targeting type 2 diabetes, researchers have identified genes that encode targets for sulfonylurea and thiazolidinedione drugs, which are now extensively used in the treatment of diabetes [13].

Mendelian Randomization (MR) methods, which utilize the principle of genetic variants being randomly assigned at conception, are widely used to explore potential causal relationships between risk factors and disease outcomes [14]. Due to the random distribution based on genetics, this approach significantly reduces the impact of confounding variables [15]. MR analysis reveals causal relationships with a precision similar to that of randomized controlled trials, with advantages including reduced risk of bias and prevention of erroneous reverse causation [14].

In the field of MR analysis for drug targets, expression quantitative trait loci (eQTLs), which are single-nucleotide polymorphisms (SNPs) associated with variations in gene expression levels, are utilized as instrumental variables (IVs) to investigate genes that may be influenced by pharmacological interventions. Particularly, cis-eQTLs located near the associated genes are often selected because they directly affect gene expression [16]. To our knowledge, this study is the first to utilize MR analysis to screen a database of genes with known drug targeting capabilities [17], aiming to identify potential drug therapy targets for treating CeD.

## 2 METHODS

### 2.1 Study Design

In this study, all data used were publicly accessible and had already been approved by the relevant institutional review boards. Consequently, this research did not require further ethical review. Specifically, we collected data for a two-sample MR analysis, using blood samples as the exposure variable, cis-eQTLs related to potential drug target genes, and genome-wide association study (GWAS) data with CeD as the outcome, aiming to explore the causal relationship between potential drug targets and CeD. Moreover, for genes with significant MR analysis results, we conducted colocalization analysis to determine whether the cis-eQTLs of these genes and CeD are influenced by the same causal variants. Finally, we applied the Summary-data-based Mendelian Randomization (SMR) technique to further confirm the impact of changes in the expression levels of these potential drug target genes on the progression of CeD. The methods used in this study are similar to those in a previously published article of ours [18], aiming to identify potential therapeutic target genes for treating CeD through rigorous analysis, and thereby contributing to the improvement of treatment and quality of life for patients with CeD.

### 2.2 Source of Data

Finan *et al*. cataloged a total of 4,479 druggable genes, referring to those that encode proteins. Due to their sequence and structural similarities with existing drug targets, these proteins could potentially serve as targets for small-molecule drugs [17]. The eQTLGen Consortium provides relatively new and comprehensive summary data on cis-eQTLs, which has significantly supported our analysis. Through our research within the eQTLGen Consortium, we identified cis-eQTL data for 2,888 of these genes, all derived from blood samples (Supplementary Table **S1**). This eQTL data helps identify genetic variants that influence gene expression levels in the blood, with these variants located no more than 1 Mb from the central gene locus. Each variant has a minor allele frequency exceeding 0.01. Genetic instruments for CeD were derived from the 11th edition of the FinnGen database, published in 2024. This database includes 4,568 celiac disease patients and 433,899 controls.

### 2.3 IVs Selection and Data Harmonization

We used SNPs as IVs for MR analysis. To ensure the reliability of our results, the included SNPs had to meet three criteria: 1. There must be a strong association between the SNP and the exposure variable; 2. The SNP should not be associated with any confounding factors; 3. The SNP should not be directly related to the outcome of the study [19]. In this research, we strictly selected SNPs based on a genome-wide significance threshold (*P <* 5 × 10^-8). To validate the instrumental strength for MR analysis, we excluded weak IVs with an F-statistic < 10, as well as palindromic or ambiguous SNPs [20, 21]. Additionally, to ensure a sufficient number of IVs for analysis, we clustered SNPs based on linkage disequilibrium using a 10,000 kb window and an r^2 threshold below 0.1 [16]. Through a comprehensive literature review, we carefully evaluated all phenotypes associated with the genetic instruments used, meticulously removing any SNPs linked to potential confounders to maintain the validity of our causal inference.

### 2.4 Preliminary Analysis - Screening of Genes

To explore the causal relationship between 2888 druggable genes and celiac disease, we conducted a two-sample MR analysis using these genes as the exposure and CeD as the outcome. Our primary analysis method was the Inverse Variance Weighted (IVW) method, and we utilized the False Discovery Rate (FDR) correction to identify potential gene targets. This strategy combines meta-analysis techniques with the Wald estimates for each SNP [22]. Additionally, we incorporated several other methods, including Bayesian Weighted Mendelian Randomization (BWMR) [23], Simple mode, Weighted Median [24], and Weighted Mode methods [25]. These methods are based on different assumptions, thereby further enhancing the reliability of our study results.

Due to variations in experimental design, demographic characteristics of the study population, and SNP variations, heterogeneity may exist in our research results. To assess this heterogeneity, we employed the IVW and MR-Egger methods. We quantified the heterogeneity of genetic instruments using Cochrane's Q statistic, where a *P-value >* 0.05 indicates minimal heterogeneity, suggesting consistent effects across different genetic tools [26]. Additionally, we conducted horizontal pleiotropy testing using the MR-Egger intercept test. A *P-value >* 0.05 indicates no significant pleiotropy, further validating the reliability of our results [27]. We also utilized the MR-PRESSO test to identify and exclude outliers in the IVW method, thereby enhancing the accuracy of our findings [28]. Concurrently, we performed a “leave-one-out” analysis to assess the impact of each SNP on the exposure-outcome relationship [29]. We also calculated the statistical power for each study and excluded any results where the power was below 80% to reduce the risk of Type II errors (https://sb452.shinyapps.io/power/) [30].

### 2.5 Colocalization Analysis

We conducted Bayesian colocalization analysis using the “coloc” R package to identify evidence of shared causal variants between gene expression and the risk of CeD. Specifically, this method utilizes Bayesian approaches to evaluate the probabilities of five mutually exclusive scenarios: (1) no association with any trait or SNP; (2) association only with trait 1; (3) association only with trait 2; (4) associations with both traits 1 and 2, but with distinct causal variants; (5) a shared causal association between traits 1 and 2. Posterior probabilities for these hypotheses, from H0 to H4, are calculated. A posterior probability (PP.H4) exceeding 0.75 indicates significant colocalization between the two traits [31].

### 2.6 SMR Analysis

SMR analysis is a statistical method based on genetic principles, used to investigate and confirm the association between genetic variants and phenotypes [32]. Combined with the complementary Heterogeneity in Dependent Instruments (HEIDI) test, SMR analysis aims to determine whether the effects of SNPs on phenotypes are mediated through changes in protein expression [32]. By conducting SMR analysis on potential drug target genes identified through colocalization analysis, we can further enhance the reliability of our research findings.

### 2.7 Statistical Analysis

For our detailed analysis, we utilized R software (version 4.2.0, http://www.r-project.org) and the “Two-Sample MR” package (version 0.5.6) to perform the MR analysis [33].

## 3 RESULTS

The workflow of our study is illustrated in Fig. (**1**). Our computational validation demonstrated that the statistical power of our MR analysis exceeded the threshold of 80%, underscoring the robustness of our findings in identifying these associations.

### 3.1 Screening for Potential Therapeutic Target Genes

To identify potential drug targets for the treatment of CeD, we utilized cis-eQTL data from 2888 druggable genes as exposure variables, with celiac disease as the outcome variable. Using the IVW method, we identified genes with an FDR < 0.05 as potential therapeutic targets. The results revealed 32 genes that could serve as potential targets for treating CeD (Supplementary Table **S2**). To enhance the credibility of our findings, we conducted tests for horizontal pleiotropy and heterogeneity on these MR analysis results, retaining 18 druggable genes that did not exhibit horizontal pleiotropy or heterogeneity (Supplementary Table **S3**). In Fig. (**2**), we present the IVW screening results of 18 druggable genes in the MR analysis of CeD, and these will be used for subsequent analysis.

### 3.2 Colocalization Analysis

**Fig. (3):**
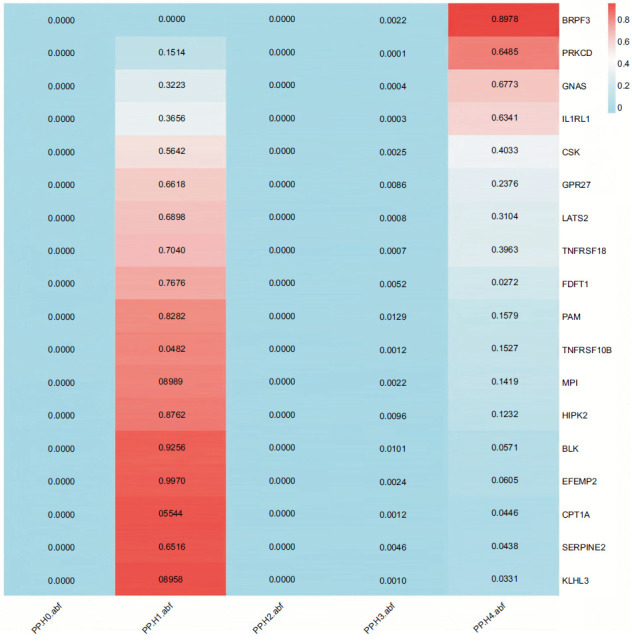
Results of the colocalization analysis between 18 druggable genes and celiac disease.

Following our initial analysis, we identified 18 potential drug target genes with significant causal links to CeD. To further strengthen the reliability of these findings, we conducted a colocalization analysis to assess whether there is a shared causal variant between the top cis-eQTLs of these genes and CeD. The analysis revealed that BRPF3 (PP.H4 = 0.998) and PRKCD (PP.H4 = 0.849) share causal variants with CeD, further validating their connection (Fig. **3**). However, the PP.H4 values for the other drug target genes were all below 0.75, indicating insufficient evidence of colocalization between these genes and the progression of CeD (Fig. **3**).

### 3.3 Validation Analysis of Different MR Methods

**Fig. (4):**
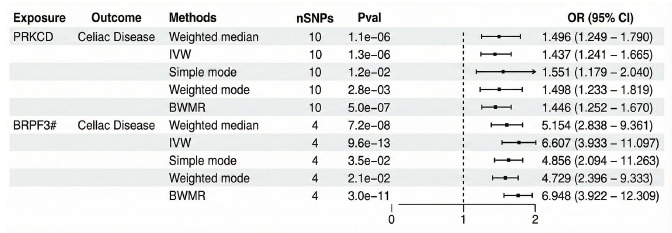
Results of MR analyses for the druggable genes PRKCD and BRPF3 in relation to CeD. **Note:** #, An unusually high OR value of 6.607 according to the IVW method suggests that this result might be less credible; **Abbreviations:** BWMR, Bayesian Weighted Mendelian Randomization; CeD, Celiac Disease; CI, Confidence Interval; IVW, Inverse Variance Weighted; MR, Mendelian Randomization; nSNPs, Number of Single Nucleotide Polymorphisms; OR, Odds Ratio.

To further validate the reliability of our MR analysis results, we employed several robust methods, including BWMR, weighted median, simple mode, and weighted mode, to verify the findings for the two genes mentioned earlier, enhancing the robustness of the results. The results from all analysis methods were consistent with those from the IVW method, further confirming the reliability of these findings (Fig. **4**). Specifically, the PRKCD gene showed a significant causal relationship with CeD (IVW: Odds Ratio (OR) 1.437, 95% Confidence Interval (CI) 1.241–1.665, *P =* 1.33E-06; Weighted Median: OR 1.496, 95% CI 1.249–1.790, *P =* 1.14E-05; Simple Mode: OR 1.551, 95% CI 1.179–2.040, *P =* 1.20E-02; Weighted Mode: OR 1.498, 95% CI 1.233–1.819, *P =* 2.77E-03; BWMR: OR 1.446, 95% CI 1.252–1.670, *P =* 4.97E-07).

However, despite a significant causal relationship indicated between the BRPF3 gene and CeD in MR analyses, the limited number of SNPs representing the BRPF3 gene and an unusually high OR value of 6.607 according to the IVW method suggest that this result might be less credible (Fig. **4**). Therefore, we decided to exclude it from further study.

### 3.4 “Leave-one-out” Sensitivity Analysis

To further validate the reliability of the MR analysis results for PRKCD as a potential therapeutic target for CeD, we conducted a “leave-one-out” sensitivity analysis. This method assesses the impact of each SNP on the exposure-outcome relationship. The result indicated that removing individual SNPs does not significantly alter the effect size or the overall interpretability of the model, as shown in Fig. (**5**).

### 3.5 SMR Analysis

In our study, through a series of analyses, the PRKCD gene has been identified as a promising drug target for the prevention and treatment of CeD. To further strengthen the reliability of our MR analysis results, we employed SMR analysis. The results of this analysis not only further confirmed the significant causal relationship between PRKCD and CeD (*P_SMR =* 4.70E-04) but also demonstrated consistency in the direction of effect. Additionally, results from the HEIDI test (*P =* 7.41E-01) showed no evidence of heterogeneity (Table **1**). This suite of analyses bolsters our confidence in PRKCD as a potential therapeutic target for treating CeD, providing a robust scientific basis for future drug development and therapeutic strategies.

## 4 DISCUSSION

CeD is a severe autoimmune disorder. Over the past 20 years, CD has emerged as a significant global public health issue [5]. However, the U.S. Food and Drug Administration has not approved any specific medications for the treatment of CeD [6]. While a gluten-free diet is effective for many, it also presents numerous challenges, including dietary restrictions, social and psychological distress, and the risk of accidental gluten exposure [34]. The development of non-dietary therapies, such as gluten chelators, transglutaminase inhibitors, and lymphocyte trafficking blockers, represents a significant advancement in treatment options [6]. Nonetheless, there remains a substantial unmet medical need for CeD-specific pharmaceutical treatments. In this study, we collected GWAS data on 2,525 druggable genes from the eQTLGen consortium and initially identified 18 potential drug targets for CeD using MR analysis. Further validation through colocalization analysis and SMR analysis confirmed that the PRKCD gene is the most promising therapeutic target for treating CeD.

The intestinal epithelium represents the largest interface between the external and internal environments, playing a crucial role [35]. It not only prevents the invasion of foreign antigens and microbes but also is responsible for the absorption of essential nutrients, water, and electrolytes [36, 37]. This process requires the precise regulation of barrier permeability through tight junctions (TJs) to maintain the balance of barrier functions [38]. Research indicated that in patients with CeD, the barrier was compromised due to abnormal paracellular transport within the intestine, which could lead to diarrhea [39]. Therefore, restoring the integrity and functionality of this barrier became a key objective in the development of medications for CeD. In this context, Larazotide acetate, a locally acting regulator of TJs aimed at reducing intestinal permeability, had become the only drug to enter Phase III development so far. However, due to potential inadequacies in the drug’s mechanism to fully address the complexity of the disease and issues related to sample size, the study was ultimately discontinued [6].

In the field of cellular biology, the Protein Kinase C (PKC) family represents a diverse group of serine/threonine kinases that play regulatory roles in various processes, including cell proliferation, differentiation, and apoptosis [40, 41]. Numerous studies have indicated that PKC is a kinase associated with the regulation of various TJ proteins, where the phosphorylation of claudins, occludin, and Zonula occludens-1 plays a pivotal role [42-44]. Specifically, PRKCD, a member of the novel PKCs, has been implicated in the pathogenesis of several autoimmune diseases, including inflammatory bowel disease and lupus [41, 45]. In our study, we found that the overexpression of PRKCD was a significant risk factor for the development of CeD and could potentially serve as a target for controlling the progression of CeD. Currently, research on the relationship between PRKCD and CeD is scarce, indicating that this area warrants further exploration.

Based on existing research, we hypothesized two possibilities. First, studies had shown that increased levels of PRKCD are closely related to changes in the composition and barrier function of TJ proteins [46]. The enhancement of the TJ complex depended on the activity of PKC [47-49]. An increase in PRKCD activity could be one of the key factors driving the upregulation of TJ protein expression and the improvement of transepithelial electrical resistance [46]. Although it is generally believed that TJ proteins can seal paracellular clefts to prevent the uncontrolled passage of solutes and water [50], extensive research has demonstrated that in various epithelial tissues, certain TJ proteins form specific paracellular channels that preferentially transport cations (such as claudin-2, claudin-10b, claudin-15), anions (such as claudin-10a, claudin-17), and water (such as claudin-2) [51-53]. Under multiple pathological conditions, the expression of barrier-forming claudin proteins may be downregulated, or the expression of channel-forming claudin proteins may be upregulated, thereby increasing selective paracellular solute transport [54]. Notably, inhibitors of PRKCD were able to reduce the expression of multiple claudin proteins [46], and these changes could lead to pathophysiological alterations in patients with celiac disease. Secondly, studies have indicated that an abnormal increase in PRKCD might lead to an over-enhancement of the composition and function of TJs, which actually might reflect an abnormal adjustment and imbalance of barrier function, rather than a normal physiological response [46]. This imbalance could limit the overall paracellular flux of ions and water. Although the barrier's electrical resistance might appear enhanced, this could actually lead to dysregulation of nutrient absorption and environmental protection functions, subsequently affecting the overall health and function of the intestine [46, 55, 56].

Our research boasts numerous advantages. First, we employed stringent criteria to select IVs and retained only those potential drug target genes with an FDR < 0.05. Additionally, we validated the reliability of our results using various methods. Secondly, to our knowledge, this study is the first to explore drug target genes for treating CeD using MR analysis of drug targets. Currently, a gluten-free diet remains the only known effective treatment strategy for CeD. There is a significant unmet medical need for effective pharmacological treatments for CeD. For individuals with genetic predispositions, early detection and monitoring of the PRKCD gene could represent a new strategy to prevent the onset of CeD, offering the possibility of personalized treatment. Furthermore, our use of drug target MR analysis has identified PRKCD as a potential drug target for treating CeD, a method that is more economical and efficient than traditional approaches.

Our study also has several limitations. First, our findings rely solely on the analysis of large datasets without empirical validation. We plan to further validate our findings through the construction of animal models in future research. Second, although we have made every effort to avoid issues such as linkage disequilibrium, pleiotropy, and heterogeneity following conventional MR analysis procedures, we cannot fully guarantee that each SNP site affects the outcome solely through the exposure. Unidentified confounding factors may influence our results. Third, our study population primarily consists of individuals of European descent, and our results may not be broadly applicable to other ethnic groups. Despite these limitations, our findings still provide important insights for further analysis of the related mechanisms.

## CONCLUSION

Currently, avoiding gluten-containing products through dietary management is the only known treatment strategy for CeD. In practice, completely avoiding gluten-containing products has proven to be highly challenging. Through MR analysis, we were the first to establish a causal relationship between PRKCD and CeD, offering fresh insights into the treatment of CeD and guiding the development of corresponding therapeutic drugs. Additionally, we further discussed the potential mechanisms of action, hypothesizing that PRKCD may alter intestinal barrier function by regulating tight junction proteins. These significant findings will form the cornerstone of our subsequent research validation efforts. It should be emphasized that since our current data primarily derives from European genomic databases, the generalizability of these conclusions to Asian and other ethnic populations requires further validation through multicenter clinical studies.

In summary, this study makes three key contributions: (1) At the scientific level, it reveals for the first time the translational potential of PRKCD signaling pathway as a novel biomarker, providing crucial theoretical foundations for developing precision diagnostic and therapeutic strategies for CeD; (2) For clinical practice, we recommend progressive clinical validation and application of PRKCD-related molecular markers to complement existing serological testing protocols; (3) Regarding patient management, while maintaining strict gluten-free dietary therapy as the cornerstone treatment, the future development and clinical application of PRKCD-targeted therapeutics may offer new hope for improving patients' quality of life and long-term prognosis.

## AUTHORS’ CONTRIBUTIONS

The authors confirm their contribution as follows: J. Z and Y. X were involved in the study concept and design; K. W, Y. X and C. C collected data and conducted analyses; J. Z wrote the draft of the article; H. W and Y. X revised the manuscript and had primary responsibility for final content. All authors gave final approval for publication and agreed to be held accountable for the work performed therein.

## Figures and Tables

**Fig. (1):**
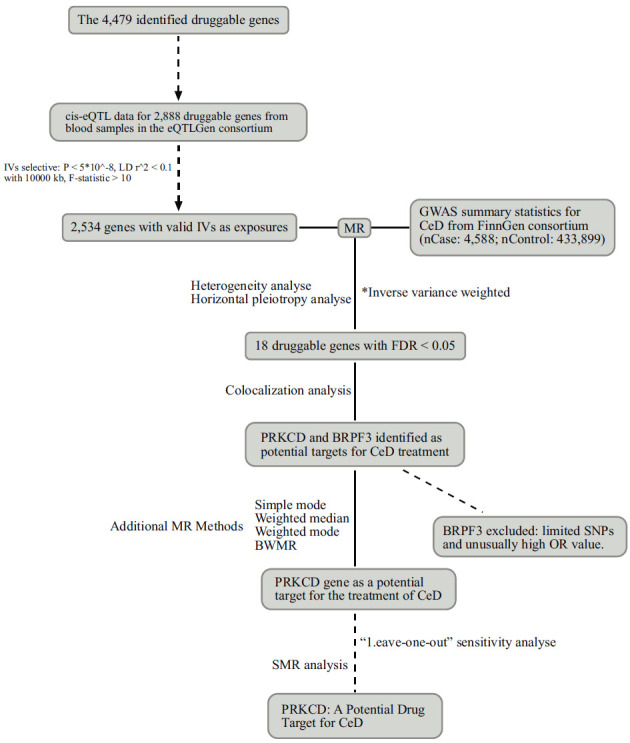
The schematic representation of the study design. **Abbreviations:** BWMR, Bayesian Weighted Mendelian Randomization; CeD, Celiac Disease; eQTLs, Expression Quantitative Trait Loci; FDR, False Discovery Rate; GWAS, Genome-wide Association Study; IVW, Inverse Variance Weighted; LD, Linkage Disequilibrium; MR, Mendelian Randomization; SMR, Summary-data-based Mendelian Randomization.

**Fig. (2):**
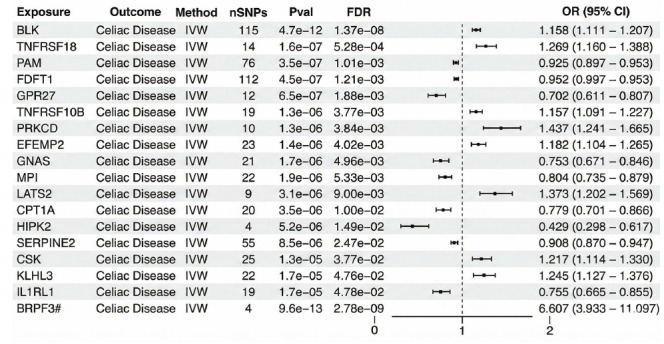
MR results linking 18 druggable genes on CeD. **Note:** #, An unusually high OR value of 6.607 according to the IVW method suggests that this result might be less credible; **Abbreviations:** BWMR, Bayesian Weighted Mendelian Randomization; CI, Confidence Interval; FDR, False Discovery Rate. IVW, Inverse Variance Weighted; MR, Mendelian Randomization; nSNPs, Number of Single Nucleotide Polymorphisms; OR, Odds Ratio.

**Fig. (5):**
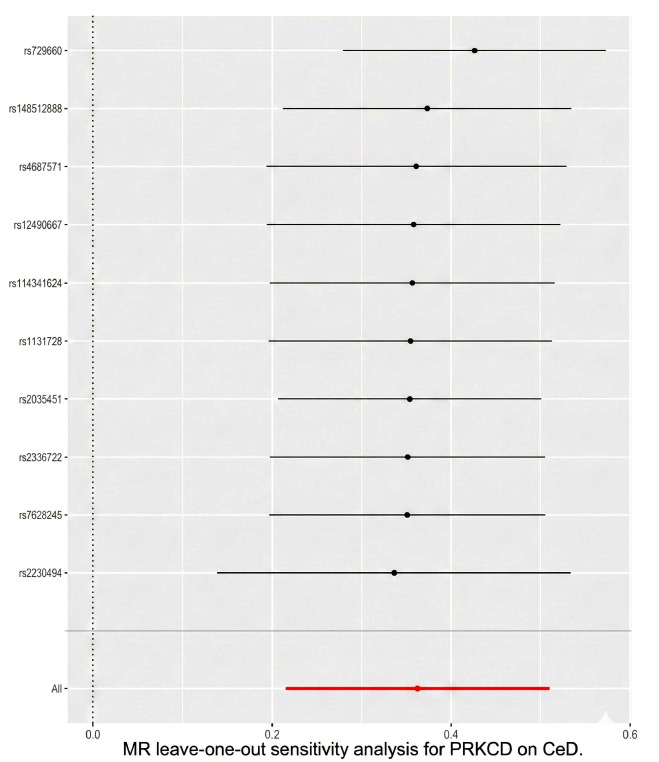
MR leave-one-out sensitivity analysis for PRKCD on CeD. **Abbreviations:** CeD, Celiac Disease; MR, Mendelian Randomization.

**Table 1:** Results of the SMR analysis for the druggable gene PRKCD.

Exposure	Outcome	b_SMR	se_SMR	p_SMR	p_HEIDI	nsnp_HEIDI
PRKCD	CeD	0.408	0.117	4.70E-04	7.41E-01	20

**Abbreviations:** SMR, Summary-data-based Mendelian Randomization; CeD, Celiac Disease.

## Data Availability

The data and supportive information are available within the article.

## LIST OF ABBREVIATIONS

CeD: Celiac Disease
GFD: Gluten-free Diet
MR: Mendelian Randomization
eQTLs: Expression Quantitative Trait Loci
SNP: Single Nucleotide Polymorphisms
IVs: Instrumental Variables
GWAS: Genome-wide Association Study
SMR: Summary-data-based Mendelian Randomization
HEIDI: Heterogeneity in Dependent Instruments
BWMR: Bayesian Weighted Mendelian Randomization
cis-eQTL: Cis-expression Quantitative Trait Loci
FDR: False Discovery Rate
TJs: Tight Junctions
PKC: Protein Kinase C

## STANDARDS FOR REPORTING

STROBE-MR guidelines were followed in this study.

## References

[1] Ludvigsson J.F., Leffler D.A., Bai J.C., Biagi F., Fasano A., Green P.H.R., Hadjivassiliou M., Kaukinen K., Kelly C.P., Leonard J.N., Lundin K.E.A., Murray J.A., Sanders D.S., Walker M.M., Zingone F., Ciacci C. (2013). The Oslo definitions for coeliac disease and related terms.. Gut..

[2] Massironi S., Franchina M., Elvevi A., Barisani D. (2024). Beyond the gluten-free diet: Innovations in celiac disease therapeutics.. World J. Gastroenterol..

[3] Rondanelli M., Faliva M.A., Gasparri C., Peroni G., Naso M., Picciotto G., Riva A., Nichetti M., Infantino V., Alalwan T.A., Perna S. (2019). Micronutrients dietary supplementation advices for celiac patients on long-term gluten-free diet with good compliance: A review.. Medicina.

[4] Stazi A.V., Trecca A., Trinti B. (2008). Osteoporosis in celiac disease and in endocrine and reproductive disorders.. World J. Gastroenterol..

[5] Aljada B., Zohni A., El-Matary W. (2021). The gluten-free diet for celiac disease and beyond.. Nutrients.

[6] Buriánek F., Gege C., Marinković P. (2024). New developments in celiac disease treatments.. Drug Discov. Today.

[7] Fernández-Bañares F., Beltrán B., Salas A., Comino I., Ballester-Clau R., Ferrer C., Molina-Infante J., Rosinach M., Modolell I., Rodríguez-Moranta F., Arau B., Segura V., Fernández-Salazar L., Santolaria S., Esteve M., Sousa C. (2021). Persistent villous atrophy in de novo adult patients with celiac disease and strict control of gluten-free diet adherence: A multicenter prospective study (CADER Study).. Am. J. Gastroenterol..

[8] Sharkey L.M., Corbett G., Currie E., Lee J., Sweeney N., Woodward J.M. (2013). Optimising delivery of care in coeliac disease – Comparison of the benefits of repeat biopsy and serological follow-up.. Aliment. Pharmacol. Ther..

[9] Clifford S., Taylor A.J., Gerber M., Devine J., Cho M., Walker R., Stefani I., Fidel S., Drahos J., Leffler D.A. (2020). Concepts and instruments for patient-reported outcome assessment in celiac disease: Literature review and experts’ perspectives.. Value Health.

[10] Lebwohl B., Granath F., Ekbom A., Smedby K.E., Murray J.A., Neugut A.I., Green P.H.R., Ludvigsson J.F. (2013). Mucosal healing and risk for lymphoproliferative malignancy in celiac disease: A population-based cohort study.. Ann. Intern. Med..

[11] Nelson M.R., Tipney H., Painter J.L., Shen J., Nicoletti P., Shen Y., Floratos A., Sham P.C., Li M.J., Wang J., Cardon L.R., Whittaker J.C., Sanseau P. (2015). The support of human genetic evidence for approved drug indications.. Nat. Genet..

[12] King E.A., Davis J.W., Degner J.F. (2019). Are drug targets with genetic support twice as likely to be approved? Revised estimates of the impact of genetic support for drug mechanisms on the probability of drug approval.. PLoS Genet.

[13] Saxena R., Voight B.F., Lyssenko V., Burtt N.P., de Bakker P.I.W., Chen H., Roix J.J., Kathiresan S., Hirschhorn J.N., Daly M.J., Hughes T.E., Groop L., Altshuler D., Almgren P., Florez J.C., Meyer J., Ardlie K., Bengtsson Boström K., Isomaa B., Lettre G., Lindblad U., Lyon H.N., Melander O., Newton-Cheh C., Nilsson P., Orho-Melander M., Råstam L., Speliotes E.K., Taskinen M.R., Tuomi T., Guiducci C., Berglund A., Carlson J., Gianniny L., Hackett R., Hall L., Holmkvist J., Laurila E., Sjögren M., Sterner M., Surti A., Svensson M., Svensson M., Tewhey R., Blumenstiel B., Parkin M., DeFelice M., Barry R., Brodeur W., Camarata J., Chia N., Fava M., Gibbons J., Handsaker B., Healy C., Nguyen K., Gates C., Sougnez C., Gage D., Nizzari M., Gabriel S.B., Chirn G.W., Ma Q., Parikh H., Richardson D., Ricke D., Purcell S. (2007). Genome-wide association analysis identifies loci for type 2 diabetes and triglyceride levels.. Science.

[14] Emdin C.A., Khera A.V., Kathiresan S. (2017). Mendelian Randomization.. JAMA.

[15] Davey Smith G., Hemani G. (2014). Mendelian randomization: Genetic anchors for causal inference in epidemiological studies.. Hum. Mol. Genet..

[16] Liu Z., Peng Z., Lin H., Zhou K., Liang L., Cao J., Huang Z., Mei J. (2024). Identifying potential drug targets for idiopathic pulmonary fibrosis: A mendelian randomization study based on the druggable genes.. Respir. Res..

[17] Finan C., Gaulton A., Kruger F.A., Lumbers R.T., Shah T., Engmann J., Galver L., Kelley R., Karlsson A., Santos R., Overington J.P., Hingorani A.D., Casas J.P. (2017). The druggable genome and support for target identification and validation in drug development.. Sci. Transl. Med..

[18] Zhou J., Xu Y., Wang H., Chen C., Wang K. (2025). CDC42: Unlocking a novel therapeutic target for primary sclerosing cholangitis through Mendelian randomization.. Am. J. Transl. Res..

[19] Burgess S., Labrecque J.A. (2018). Mendelian randomization with a binary exposure variable: Interpretation and presentation of causal estimates.. Eur. J. Epidemiol..

[20] Burgess S., Thompson S.G. (2011). Avoiding bias from weak instruments in Mendelian randomization studies.. Int. J. Epidemiol..

[21] Bowden J., Del Greco  M.F., Minelli C., Davey Smith G., Sheehan N.A., Thompson J.R. (2016). Assessing the suitability of summary data for two-sample Mendelian randomization analyses using MR-Egger regression: The role of the I2 statistic.. Int. J. Epidemiol..

[22] Burgess S., Butterworth A., Thompson S.G. (2013). Mendelian randomization analysis with multiple genetic variants using summarized data.. Genet. Epidemiol..

[23] Zhao J., Ming J., Hu X., Chen G., Liu J., Yang C. (2020). Bayesian weighted Mendelian randomization for causal inference based on summary statistics.. Bioinformatics.

[24] Bowden J., Davey Smith G., Haycock P.C., Burgess S. (2016). Consistent estimation in mendelian randomization with some invalid instruments using a weighted median estimator.. Genet. Epidemiol..

[25] Hartwig F.P., Davey Smith G., Bowden J. (2017). Robust inference in summary data Mendelian randomization *via* the zero modal pleiotropy assumption.. Int. J. Epidemiol..

[26] Bowden J., Hemani G., Davey Smith G. (2018). Invited commentary: Detecting individual and global horizontal pleiotropy in mendelian randomization—A job for the humble heterogeneity statistic?. Am. J. Epidemiol..

[27] van Kippersluis H., Rietveld C.A. (2018). Pleiotropy-robust Mendelian randomization.. Int. J. Epidemiol..

[28] Chen L., Yang H., Li H., He C., Yang L., Lv G. (2022). Insights into modifiable risk factors of cholelithiasis: A Mendelian randomization study.. Hepatology.

[29] Hong J., Qu Z., Ji X., Li C., Zhang G., Jin C., Wang J., Zhang Y., Shen Y., Meng J., Zhou C., Fang C., Wang W., Yan S. (2021). Genetic associations between IL-6 and the development of autoimmune arthritis are gender-specific.. Front Immunol..

[30] Burgess S. (2014). Sample size and power calculations in Mendelian randomization with a single instrumental variable and a binary outcome.. Int. J. Epidemiol..

[31] Luo P., Yu X. (2024). ENPP2 /Autotaxin: The potential drug target for alcoholic liver disease identified through Mendelian randomization analysis.. Liver Int..

[32] Wu Y., Zeng J., Zhang F., Zhu Z., Qi T., Zheng Z., Lloyd-Jones L.R., Marioni R.E., Martin N.G., Montgomery G.W., Deary I.J., Wray N.R., Visscher P.M., McRae A.F., Yang J. (2018). Integrative analysis of omics summary data reveals putative mechanisms underlying complex traits.. Nat. Commun..

[33] Broadbent J.R., Foley C.N., Grant A.J., Mason A.M., Staley J.R., Burgess S. (2020). Mendelian Randomization v0.5.0: Updates to an R package for performing Mendelian randomization analyses using summarized data.. Wellcome Open Res..

[34] Ghunaim M., Seedi A., Alnuman D., Aljohani S., Aljuhani N., Almourai M., Alsuhaymi S. (2024). Impact of a gluten-free diet in adults with celiac disease: Nutritional deficiencies and challenges.. Cureus.

[35] Macura B., Kiecka A., Szczepanik M. (2024). Intestinal permeability disturbances: Causes, diseases and therapy.. Clin. Exp. Med..

[36] Groschwitz K.R., Hogan S.P. (2009). Intestinal barrier function: Molecular regulation and disease pathogenesis.. J. Allergy Clin. Immunol..

[37] Celebi Sozener Z., Ozdel Ozturk B., Cerci P., Turk M., Gorgulu Akin B., Akdis M., Altiner S., Ozbey U., Ogulur I., Mitamura Y., Yilmaz I., Nadeau K., Ozdemir C., Mungan D., Akdis C.A. (2022). Epithelial barrier hypothesis: Effect of the external exposome on the microbiome and epithelial barriers in allergic disease.. Allergy.

[38] Varadarajan S., Stephenson R.E., Miller A.L. (2019). Multiscale dynamics of tight junction remodeling.. J. Cell Sci..

[39] Schumann M., Siegmund B., Schulzke J.D., Fromm M. (2017). Celiac Disease: Role of the epithelial barrier.. Cell Mol. Gastroenterol. Hepatol..

[40] Steinberg S.F. (2008). Structural basis of protein kinase C isoform function.. Physiol. Rev..

[41] Chavan S.V., Desikan S., Roman C.A.J., Huan C. (2024). PKCδ protects against lupus autoimmunity.. Biomedicines.

[42] Franke A., McGovern D.P.B., Barrett J.C., Wang K., Radford-Smith G.L., Ahmad T., Lees C.W., Balschun T., Lee J., Roberts R., Anderson C.A., Bis J.C., Bumpstead S., Ellinghaus D., Festen E.M., Georges M., Green T., Haritunians T., Jostins L., Latiano A., Mathew C.G., Montgomery G.W., Prescott N.J., Raychaudhuri S., Rotter J.I., Schumm P., Sharma Y., Simms L.A., Taylor K.D., Whiteman D., Wijmenga C., Baldassano R.N., Barclay M., Bayless T.M., Brand S., Büning C., Cohen A., Colombel J.F., Cottone M., Stronati L., Denson T., De Vos M., D’Inca R., Dubinsky M., Edwards C., Florin T., Franchimont D., Gearry R., Glas J., Van Gossum A., Guthery S.L., Halfvarson J., Verspaget H.W., Hugot J.P., Karban A., Laukens D., Lawrance I., Lemann M., Levine A., Libioulle C., Louis E., Mowat C., Newman W., Panés J., Phillips A., Proctor D.D., Regueiro M., Russell R., Rutgeerts P., Sanderson J., Sans M., Seibold F., Steinhart A.H., Stokkers P.C.F., Torkvist L., Kullak-Ublick G., Wilson D., Walters T., Targan S.R., Brant S.R., Rioux J.D., D’Amato M., Weersma R.K., Kugathasan S., Griffiths A.M., Mansfield J.C., Vermeire S., Duerr R.H., Silverberg M.S., Satsangi J., Schreiber S., Cho J.H., Annese V., Hakonarson H., Daly M.J., Parkes M. (2010). Genome-wide meta-analysis increases to 71 the number of confirmed Crohn’s disease susceptibility loci.. Nat. Genet..

[43] Suzuki T., Elias B.C., Seth A., Shen L., Turner J.R., Giorgianni F., Desiderio D., Guntaka R., Rao R. (2009). PKCη regulates occludin phosphorylation and epithelial tight junction integrity.. Proc. Natl. Acad. Sci. USA.

[44] Cario E., Gerken G., Podolsky D.K. (2004). Toll-like receptor 2 enhances ZO-1-associated intestinal epithelial barrier integrity *via* protein kinase C.. Gastroenterology.

[45] Altman A., Kong K.F. (2014). Protein kinase C inhibitors for immune disorders.. Drug Disco. Today.

[46] Segui-Perez C., Stapels D.A.C., Ma Z., Su J., Passchier E., Westendorp B., Wubbolts R.W., Wu W., van Putten J.P.M., Strijbis K. (2024). MUC13 negatively regulates tight junction proteins and intestinal epithelial barrier integrity *via* protein kinase C.. J. Cell Sci..

[47] Stuart R.O., Nigam S.K. (1995). Regulated assembly of tight junctions by protein kinase C.. Proc. Natl. Acad. Sci. USA.

[48] Yoo J., Nichols A., Mammen J., Calvo I., Song J.C., Worrell R.T., Matlin K., Matthews J.B. (2003). Bryostatin-1 enhances barrier function in T84 epithelia through PKC-dependent regulation of tight junction proteins.. Am. J. Physiol. Cell Physiol..

[49] Koizumi J., Kojima T., Ogasawara N., Kamekura R., Kurose M., Go M., Harimaya A., Murata M., Osanai M., Chiba H., Himi T., Sawada N. (2008). Protein kinase C enhances tight junction barrier function of human nasal epithelial cells in primary culture by transcriptional regulation.. Mol. Pharmacol..

[50] Turner J.R. (2009). Intestinal mucosal barrier function in health and disease.. Nat.Rev. Immunol..

[51] Amasheh S., Meiri N., Gitter A.H., Schöneberg T., Mankertz J., Schulzke J.D., Fromm M. (2002). Claudin-2 expression induces cation-selective channels in tight junctions of epithelial cells.. J. Cell Sci..

[52] Günzel D., Stuiver M., Kausalya P.J., Haisch L., Krug S.M., Rosenthal R., Meij I.C., Hunziker W., Fromm M., Müller D. (2009). Claudin-10 exists in six alternatively spliced isoforms that exhibit distinct localization and function.. J. Cell Sci..

[53] Krug S.M., Günzel D., Conrad M.P., Rosenthal R., Fromm A., Amasheh S., Schulzke J.D., Fromm M. (2012). Claudin-17 forms tight junction channels with distinct anion selectivity.. Cell Mol. Life Sci..

[54] Krug S.M., Schulzke J.D., Fromm M. (2014). Tight junction, selective permeability, and related diseases.. Semin. Cell Dev. Biol..

[55] Wada M., Tamura A., Takahashi N., Tsukita S. (2013). Loss of claudins 2 and 15 from mice causes defects in paracellular Na+ flow and nutrient transport in gut and leads to death from malnutrition.. Gastroenterology.

[56] Tsai P.Y., Zhang B., He W.Q., Zha J.M., Odenwald M.A., Singh G., Tamura A., Shen L., Sailer A., Yeruva S., Kuo W.T., Fu Y.X., Tsukita S., Turner J.R. (2017). IL-22 upregulates epithelial claudin-2 to drive diarrhea and enteric pathogen clearance.. Cell Host Micro..

